# Sudden unexpected infant death (SUDI) in a newborn due to medium chain acyl CoA dehydrogenase (MCAD) deficiency with an unusual severe genotype

**DOI:** 10.1186/1824-7288-38-59

**Published:** 2012-10-24

**Authors:** Cristina Lovera, Francesco Porta, Anna Caciotti, Serena Catarzi, Michela Cassanello, Ubaldo Caruso, Maria Rita Gallina, Amelia Morrone, Marco Spada

**Affiliations:** 1 Department of Pediatrics, University of Torino, Regina Margherita Children’s Hospital, Piazza Polonia 94, Torino, 10126, Italy; 2 Metabolic and Muscular Unit, Clinic of Pediatric Neurology, Meyer Childrens’Hospital, Florence, Italy; 3 Department of Sciences for Woman and Child's Health, University of Florence, Florence, Italy; 4 Laboratory for the Study of Inborn Errors of Metabolism, - University Department of Pediatrics, G. Gaslini Institute, Genoa, Italy; 5 Newborn Intensive Care Unit, Maggiore della Carità Hospital, Novara, Italy

**Keywords:** Medium chain acyl CoA dehydrogenase deficiency, Sudden unexpected deaths in Infancy, Sudden infant death syndrome, Fatty acid oxidation disorders

## Abstract

Medium chain acyl CoA dehydrogenase deficiency (MCAD) is the most common inborn error of fatty acid oxidation. This condition may lead to cellular energy shortage and cause severe clinical events such as hypoketotic hypoglycemia, Reye syndrome and sudden death. MCAD deficiency usually presents around three to six months of life, following catabolic stress as intercurrent infections or prolonged fasting, whilst neonatal-onset of the disease is quite rare. We report the case of an apparently healthy newborn who suddenly died at the third day of life, in which the diagnosis of MCAD deficiency was possible through peri-mortem blood-spot acylcarnitine analysis that showed very high concentrations of octanoylcarnitine. Genetic analysis at the ACADM locus confirmed the biochemical findings by demonstrating the presence in homozygosity of the frame-shift c.244dup1 (p.Trp82LeufsX23) mutation, a severe genotype that may explain the unusual and very early fatal outcome in this newborn. This report confirms that inborn errors of fatty acid oxidation represent one of the genetic causes of sudden unexpected deaths in infancy (SUDI) and underlines the importance to include systematically specific metabolic screening in any neonatal unexpected death.

## Background

Specific genetic conditions may lead to sudden unexpected deaths in infancy (SUDI), such as inborn errors of fatty acid oxidation and genetic disorders of cardiac ion channels [1]. Medium chain acyl CoA dehydrogenase (MCAD) deficiency (OMIM 201450) is an autosomal recessive disorder and represents the most common fatty acid oxidation disorder with an incidence around 1:8,000 [2]. The disease may present dramatically with severe hypoketotic hypoglycemia, Reye syndrome or sudden death, typically with a peak of frequency around 3–6 month, whilst neonatal SUDI is quite rare. When undetected, approximately 20–25% of infants will die or suffer permanent neurologic impairment as a consequence of the first acute metabolic decompensation. Detection of MCAD deficiency through newborn screening, however, has allowed counseling of parents and primary care to prevent fasting stress in pre-symptomatic infants [3]. Here we report the case of a newborn affected by MCAD deficiency and homozygous for a severe genetic mutation who suddenly died at the third day of life.

## Case presentation

The child was born by spontaneous delivery at 40th week of gestation after normal pregnancy from healthy consanguineous parents. APGAR score was 8/9 and anthropometric parameters were within the normal percentiles.

On third day of life, when discharge was already scheduled, the baby had sudden cardiac-respiratory arrest and immediate resuscitation was performed. Despite prompt resuscitation the patient died in the following hours because of irreversible multi-organ failure.

First laboratory investigation revealed profound hypoglycaemia (0.5 mmol/l) and mild hyperammonemia (176 μmol/l). Blood spot and urine collection allowed specific metabolic investigation that showed very marked elevation of medium chain acylcarnitines as hexanoyl carnitine (C6), (blood spot concentration at 1.75 μmol/L; normal value <0.20), octanoyl carnitine (C8), (blood spot concentration at 36.74 μmol/L; normal value < 0.23), decenoyl carnitine (C10), (blood spot concentration at 1.74 μmol/L; normal value <0.22), in association with high urinary excretion of suberylglycine (7.7 μMol/mMol Creatinine; normal value <2) and exanoylglycine (4.8 μMol/mMol Creatinine; normal value <2). These metabolic findings were formally consistent with the diagnosis of MCAD deficiency. In order to confirm the diagnosis at molecular level, the genomic DNA was isolated from a whole sample blood collected and informed consent for genetic testing obtained from parents.

Mutation analysis of *ACADM* gene revealed homozygosity for the c.244dup1 (p.Trp82LeufsX23) single base duplication. This mutation leads to a severe frame-shift with the introduction of a premature stop codon. Both parents were heterozygous for such mutation at molecular analysis.

## Discussion

Sudden infant death syndrome (SIDS) is a multifactorial disorder influenced by developmental, environmental, and biological risk factors and it is defined as the sudden death of an infant that is unexpected by history and unexplained by postmortem examinations. SIDS is a leading cause of infant mortality, accounting for 8% of all infant deaths [4] and it is the most common cause of post neonatal infant mortality accounting for 40-50% of all deaths between one month and one year of age. Cases in which instead the cause is identified should be diagnosed not as SIDS but as SUDI, such as in specific genetic conditions. At present, long QT syndrome and fatty acid β-oxidation disorders represent the most frequent inherited causes of SUDI [1].

Long QT syndrome is caused by mutations in genes encoding cardiac ion channels (such as KVLQT1, HERG, KCNE1, and KCNE2) leading to prolonged cardiac action potential by either increasing depolarization or decreasing repolarization current and so causing syncope, seizures or sudden death [1].

Fatty acid β-oxidation disorders encompass almost 20 different inborn enzymopathies. Among them, medium chain acyl CoA dehydrogenase (MCAD) deficiency is largely the most frequent condition with incidence around 1: 8,000 [2]. The enzymatic defect results in a blockade of medium chain fatty acids breakdown and of ketone production. The enzymatic deficiency compromises the availability of ketones during prolonged fasting or acute illness. This energy shortage primarily affects function of skeletal and cardiac muscle as well as the brain, and can lead to death. MCAD deficiency is usually silent and generally affects individuals clinically normal until an intercurrent illness episode triggers hypoglycemia and fatty acids discharge [5].

The most common clinical presentations of the disease include hypoketotic hypoglycemia, Reye syndrome and, more dramatically, sudden unexpected death, generally during the first year of life. Surviving patients who comply with a treatment regimen, which essentially relies on avoidance of fasting, either dramatically reduce or completely eliminate recurrent life-threatening disease episodes [6]. The biochemical diagnosis is based on acylcarnitine profile, characterized by accumulation of hexanoyl-carnitine (C6) to decanoyl-carnitine (C10) species, with prominent octanoyl-carnitine (C8). Patients also show a characteristic urinary organic acids pattern, with typical dicarbossilic aciduria and elevated hexanoyl and suberylglycine. The diagnosis can be confirmed by molecular analysis of *ACADM* gene (1p31.1) which encodes for MCAD protein [7]. To date more then 90 mutations have been reported in the *ACADM* gene (HGMD - Human Genome Mutation Database) [8].

The c.985A>G missense mutation is the most frequent molecular lesion and may account up to 90% of alleles of clinically diagnosed MCAD deficient patients, but is lower in cases ascertained by screening [9]. This suggests that this mutation may be more likely to lead to a severe clinical phenotype [9,10]. Although there is no “safe” genotype or metabolite profile for MCAD deficiency, profoundly elevated C8 on blood spot or the presence of severe mutations may reflect impaired ability to tolerate metabolic stress potentially leading to more severe or lethal phenotypes as confirmed in the MCAD deficient patient here reported who presented with neonatal SUDI.

Sudden neonatal death represents an unusual clinical presentation of MCAD deficiency, possibly related to specific severe genotypes, such as the homozygosity for the frame-shift c.244dup1(p.Trp82LeufsX23) mutation [11]. Unfortunately, neonatal death due to MCAD deficiency could be not prevented by routine newborn screening programs, as sampling for blood spot acylcarnitine analysis is generally carried out after 48 hours of life and results might not be available before the onset of acute clinical events. Nevertheless, expecially in those States in which expanded metabolic newborn screening is not yet available, to provide definite diagnosis and accurate genetic counseling in any sudden neonatal death, fatty acid oxidation inborn disorders must be routinely explored by collecting simple biological material as blood spot, that may be easily sent to specialized laboratories for acylcarnitine and/or DNA analysis.

## Consent

Written informed consent was obtained from the parents of the patient for publication of this case report.

## Abbreviations

MCAD: Medium chain acyl CoA dehydrogenase; SUDI: Sudden Unexpected Deaths in Infancy; SIDS: Sudden infant death syndrome; FAO: Fatty Acid Oxidation.

## Competing interests

The authors declare that they have no competing interests.

## Authors’ contributions

CL, FP, AM, MS: conception and design, acquisition of data, analysis and interpretation of data, drafting the manuscript. AC, SC, MC, UC, MRG: conception and design, analysis and interpretation of data, critical revision of the manuscript. All authors read and approved the final manuscript.
